# Longitudinal MRI and ^1^H-MRS study of SCA7 mouse forebrain reveals progressive multiregional atrophy and early brain metabolite changes indicating early neuronal and glial dysfunction

**DOI:** 10.1371/journal.pone.0296790

**Published:** 2024-01-16

**Authors:** Jean-Baptiste Pérot, Anna Niewiadomska-Cimicka, Emmanuel Brouillet, Yvon Trottier, Julien Flament

**Affiliations:** 1 Laboratoire des Maladies Neurodégénératives, Université Paris-Saclay, Commissariat à l’Energie Atomique et aux Energies Alternatives, Centre National de la Recherche Scientifique, Molecular Imaging Research Center, Fontenay-aux-Roses, 92260, France; 2 Institut du Cerveau–Paris Brain Institute–ICM, Sorbonne Université, Paris, 75013, France; 3 Institut de Génétique et de Biologie Moléculaire et Cellulaire, Illkirch, 67404, France; 4 Centre National de la Recherche Scientifique, Unité Mixte de Recherche 7104, Illkirch, 67404, France; 5 Institut National de la Santé et de la Recherche Médicale, U964, Illkirch, 67404, France; 6 Université de Strasbourg, Illkirch, 67404, France; Nathan S Kline Institute, UNITED STATES

## Abstract

SpinoCerebellar Ataxia type 7 (SCA7) is an inherited disorder caused by CAG triplet repeats encoding polyglutamine expansion in the ATXN7 protein, which is part of the transcriptional coactivator complex SAGA. The mutation primarily causes neurodegeneration in the cerebellum and retina, as well as several forebrain structures. The SCA7^140Q/5Q^ knock-in mouse model recapitulates key disease features, including loss of vision and motor performance. To characterize the temporal progression of brain degeneration of this model, we performed a longitudinal study spanning from early to late symptomatic stages using high-resolution magnetic resonance imaging (MRI) and *in vivo* ^1^H-magnetic resonance spectroscopy (^1^H-MRS). Compared to wild-type mouse littermates, MRI analysis of SCA7 mice shows progressive atrophy of defined brain structures, with the striatum, thalamus and cortex being the first and most severely affected. The volume loss of these structures coincided with increased motor impairments in SCA7 mice, suggesting an alteration of the sensory-motor network, as observed in SCA7 patients. MRI also reveals atrophy of the hippocampus and anterior commissure at mid-symptomatic stage and the midbrain and brain stem at late stage. ^1^H-MRS of hippocampus, a brain region previously shown to be dysfunctional in patients, reveals early and progressive metabolic alterations in SCA7 mice. Interestingly, abnormal glutamine accumulation precedes the hippocampal atrophy and the reduction in myo-inositol and total N-acetyl-aspartate concentrations, two markers of glial and neuronal damage, respectively. Together, our results indicate that non-cerebellar alterations and glial and neuronal metabolic impairments may play a crucial role in the development of SCA7 mouse pathology, particularly at early stages of the disease. Degenerative features of forebrain structures in SCA7 mice correspond to current observations made in patients. Our study thus provides potential biomarkers that could be used for the evaluation of future therapeutic trials using the SCA7^140Q/5Q^ model.

## Introduction

Spinocerebellar ataxias consist of a group of autosomal dominant degenerative disorders defined by progressive onset of motor symptoms such as abnormal gait and coordination. Among this class of ataxias, spinocerebellar ataxia type 7 (SCA7) is one of the less studied, due to noticeably low prevalence of <1 out of 100,000 [1]. SCA7 is caused by the abnormal expansion of CAG triplet repeats encoding a polyglutamine (polyQ) tract in ATAXIN-7 (ATXN7), and therefore belongs to the group of CAG/polyQ expansion disorders, which includes Huntington’s disease (HD) and other SCAs (SCA1-3, 6 and 17). Whereas the number of CAG repeats ranges from 4 to 35 in the *ATXN7* gene in normal individuals, the pathological mutation is typically characterized by a higher number of triplets (from 36 to 70) and causes adult-onset of the symptoms. Larger CAG expansions, which can reach more than 400 triplets [2], are associated with earlier onset and more severe and rapid disease progression [3].

The classical clinical signs of SCA7 are gait ataxia, dysarthria, dysphagia and visual impairment, which are related to the progressive atrophy of the cerebellum, brain stem and retina. However, additional volume loss has been reported in several regions of the cerebral cortex including parahippocampal gyrus, cingulate, and motor cortices [4], and likely accounts for diverse non-cerebellar symptoms that affect patients [3]. Symptoms appear after a prodromal phase, duration of which is related to the number of CAG repeats, with typical onset at the age of 30 years [5]. The disease is then progressing until death with great heterogeneity in severity and rapidity between patients. Interestingly, while shorter expansions associated with adult onset lead to ataxia as the first symptoms, large repeat expansions associated with early disease onset cause visual loss before cerebellar ataxia [3].

ATXN7 is a subunit of the Spt-Ada-Gcn5 acetyltransferase (SAGA) multiprotein complex, a co-activator of RNA polymerase II transcription, and is involved in the regulation of a large number of genes. Expansion of the polyglutamine tract likely affects the conformation of ATXN7, which accumulates in nuclei and interacts aberrantly with its protein partners, ultimately producing toxicity to neurons. The mechanisms underlying the toxicity of mutant ATXN7 are not fully understood but there is evidence that alteration of transcription, mitochondrial defects and perturbation of autophagy play a central role in ATXN7-induced neurodegeneration [6].

Over the past years, diverse genetic murine models have proven very useful to investigate the SCA7 pathology. In particular, the knock-in mouse model carrying 266 CAG repeats inserted into the murine *Atxn7* gene should best translate the human pathology [7]. Although this SCA7^266Q/5Q^ model indeed developed retinal, cerebellar and hippocampal pathology, its extensively elongated expansion leads to early death, which limits the phenotype characterization. A cognate SCA7^140Q/5Q^ knock-in line, which was derived from SCA7^266Q/5Q^ mice by spontaneous germline CAG contraction, has prolonged survival up to about 50 weeks of age and appears to more validly recapitulate the timeline and broad spectrum of SCA7 pathology [8]. SCA7^140Q/5Q^ mice present early visual impairment and progressive motor symptoms, including hypoactivity as early as 16 weeks of age and gait impairments appearing at 20–25 weeks. Neuropathology revealed modest morphological alteration of the cerebellum until 34–38 weeks of age, when Purkinje cells showed cellular shrinkage and electrophysiological dysfunction. Whole brain volume showed atrophy by 25 weeks, as measured by postmortem MRI, and appeared to coincide with the onset of motor impairment. However, region-specific brain alterations are still poorly characterized.

There are a limited number of methods allowing *in vivo* follow up of disease onset and progression and understanding of pathophysiological pathways. Alteration in the volume of brain structures measured by MRI is currently the best biomarker of disease progression in polyQ diseases [9,10]. In addition to the anatomical information provided by high-resolution MRI, in vivo ^1^H-Magnetic Resonance Spectroscopy (^1^H-MRS) can provide critical information about pathological molecular processes related to cellular metabolism in SCAs, especially SCA7 [11]. MRS allows measurement of the concentration of key metabolites such as total N-acetyl-aspartate (tNAA), glutamine (Gln), glutamate (Glu), myo-inositol (Ins) and choline (Cho). In particular, [tNAA] can be informative about neuronal health while [Gln] and [Cho] are more indicative of glial metabolism [12]. MRS was able to detect early metabolic changes in multiple SCAs, including SCA7 [11]. However, there is yet no longitudinal MRI or MRS data available for the SCA7^140Q/5Q^ mouse model, while such neurochemical MRS studies have been performed in different mouse models of SCA1 [13], SCA3 [14], and HD [15,16].

In order to further characterize *in vivo* the brain pathology in the SCA7^140Q/5Q^ knock-in model, we performed longitudinal follow-up of animal cohorts using volumetric measurements based on high-resolution anatomical MRI and brain metabolites measured by ^1^H-MRS. Based on our previous ex vivo study of this model [8], and on reports of electrophysiological defects of the hippocampal neurons [7], we hypothesized that the hippocampus could undergo early metabolic and structural modifications and thus provide a better understanding of the pathophysiology involved in this novel mouse model. We showed progressive atrophy of multiple forebrain structures as well as progressive metabolic alterations in the hippocampus. These data may prove useful to further investigate the role of non-cerebellar brain structures in the development of the pathology and the biological pathways affected by polyQ-expanded ATXN7. In addition, temporal metabolic defects and structural atrophies could be used as reliable biomarkers to monitor disease onset and progression and evaluate therapeutic interventions, particularly those using gene-silencing approaches.

## Materials & methods

### SCA7^140Q/5Q^ mouse model

SCA7^140Q/5Q^ knock-in mice harboring 140 CAG repeats inserted in the murine *Atxn7* gene were used [8]. SCA7^140Q/5Q^ mice (n = 5) were compared to their relative age-matched wild-type littermates (n = 9). Based on the current literature, SCA7 mouse pathology develops similarly in both sexes [7,8]. Therefore, for practical reasons related to animal availability, only females were used in this study. Mice were housed in a temperature-controlled room maintained on a 12 hours light/dark cycle. Food and water were available ad libitum. All animal studies were conducted according to the French regulation (EU Directive 2010/63/EU–French Act Rural Code R 214–87 to 126). The animal facility was approved by veterinarian inspectors (authorization n° A 92-032-02) and complies with Standards for Humane Care and Use of Laboratory Animals of the Office of Laboratory Animal Welfare (OLAW–n°#A5826-01). All procedures received approval from the ethical committee (APAFIS #21335–201907031642584 v2).

### MRI and ^1^H-MRS

Animals were scanned longitudinally (16, 24 and 30 weeks of age) on a horizontal 11.7 T Bruker scanner (Bruker, Ettlingen, Germany) running with ParaVision 6.0.1 software. Mice were first anesthetized using 3% isoflurane in a 1:1 gas mixture of air/O_2_ and positioned in a dedicated stereotaxic frame with mouth and ear bars to prevent any movements during MR acquisitions. Mice temperature was monitored with a rectal probe and maintained at 37°C with regulated water flow. Respiratory rate was continuously monitored using PC SAM software (Small Animal Instruments, Inc., Stony Brook, NY, USA) during scanning. The isoflurane level was adjusted around 1.5% to keep the respiratory rate in the range of 60 to 80 per minute. A quadrature cryoprobe (Bruker, Ettlingen, Germany) was used for radiofrequency transmission and reception.

High-resolution anatomical images (Turbo Spin Echo sequence, TE/TR = 5/10000 ms, Turbo factor = 10, effective TE = 45 ms, in-plane resolution = 100 x 100 μm^2^, 200 μm slice thickness, 100 slices, 20 min) were used for structures volumetry. ^1^H-MRS (LASER with VAPOR water-suppression, TE/TR = 20/5000 ms, 96 averages, 8min) was then acquired in a single voxel (dimensions 6 x 1.5 x 2 mm^3^) placed in the dorsal hippocampus. Both hemispheres of the hippocampus were included to acquire signal in a larger voxel for increased SNR. Prior to spectral acquisition, the MAPSHIM routine was performed in the same voxel to achieve good shimming (FWHM of the unsuppressed water peak was systematically lower than 15 Hz).

### Data processing and statistical analyses

Images were co-registered and automatically segmented using an in-house python library Sammba-MRI [17], as already described [18]. Metabolites concentration were estimated with LCModel [19] using default pipeline, and was normalized by total creatine (tCr). In respect of the interpolation quality criterion (Cramer-Rao lower bound ≤ 5%), several metabolites were quantified, including glutamate (Glu), glutamine (Gln), total N-acetyl-aspartate (tNAA), total choline (tCho), myo-inositol (Ins), Taurine (Tau), and gamma-aminobutyric acid (GABA). The same basis set was used for metabolites quantification of both groups, including a macromolecular basis that was acquired in wild-type mice from a previous work in the laboratory. Following ^1^H-MRS acquisition, one wild-type mouse was excluded from further analysis due to phenotype of sporadic portosystemic shunting, which is common in a substet of C57BL/6 mice [20].

Variation of volume between WT littermates and SCA7^140Q/5Q^ mice was calculated in each brain region as follows: Variation = 100 x (Volume(WT)—Volume(SCA7^140Q/Q^)) / Volume(WT).

Statistical analyses were performed using GraphPad Prism 8.0 (GraphPad Software, San Diego, California, USA). The Shapiro-Wilk test was used to test the data for normality and no deviation from normality was observed for any data. As ANOVA was not available due to missing values at 30 weeks due to death of one mouse, data were analyzed by fitting a mixed model taking repeated measures into account. The significant threshold was set to 0.05. Mixed model was followed by Sidak’s multiple comparisons test to determine significant differences between groups.

## Results

### SCA7^140Q/5Q^ mice show progressive atrophy of selected brain structures

**Fig 1:**
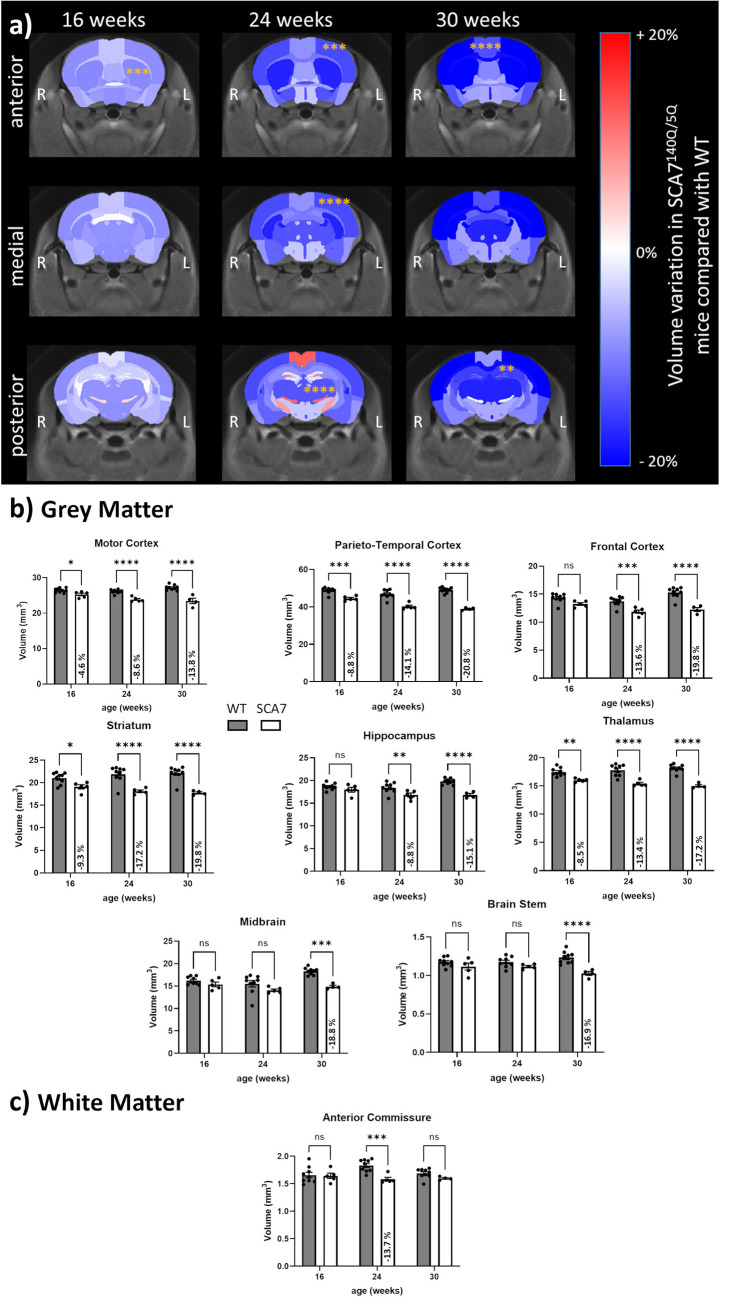
Brain structures volume measured by anatomical MRI in SCA7^140Q/5Q^ and WT mice. **a)** Volume variation maps between SCA7^140Q/5Q^ and WT mice were calculated for each structure as (Volume(SCA7)-Volume(WT))/Volume(WT). Orange stars represent significant effect of genotype on structures’ volume. **b)** Volume of structures of interest composed mainly of gray matter measured at 16, 24 and 30 weeks in WT (gray) and SCA7^140Q/5Q^ (white) mice. **c)** Volume of structures of interest composed mainly of white matter measured at 16, 24 and 30 weeks in WT (gray) and SCA7^140Q/5Q^ (white) mice. The intensity of the variation expressed in percentage was reported in the white bar when it was statistically significant. Mean ± SD, n = 9 WT, n = 5 SCA7. *p<0.05, **p<0.01, ***p<0.001, ****p<0.0001 (Mixed model + Sidak’s multiple comparisons test).

The volume of brain structures was calculated based on automatic segmentation of anatomical images and compared between SCA7^140Q/5Q^ and WT mice. The change in volume for each region was computed for each timepoint and represented as variation maps (Fig 1A). A significant effect of genotype and time was found with linear mixed model on whole brain volume and volumes of all regions presented in Fig 1B and 1C. Post-hoc multiple comparisons revealed that significant volume loss was already measured at 16 weeks gray matter structures such as the motor cortex (-4.6%, p<0.05), parieto-temporal cortex (-8.8%, p<0.001), striatum (-9.3%, p<0.05) and thalamus (-8.5%, p<0.01) of SCA7 mice. Atrophy of these brain structures was even more pronounced at 24 weeks (motor (-8.6%, p<0.0001), parieto-temporal cortex (-14.1%, p<0.0001), striatum (-17.2%, p<0.0001), thalamus (-13.4%, p<0.0001). At 24 weeks, significant atrophy was also measured in the hippocampus (-8.8%, p<0.01) and frontal (-13.6%, p<0.001) cortices, accounting for a global atrophy of the SCA7^140Q/5Q^ mouse brain (-4.7%, p<0.05). A significant atrophy was also measured in white matter, especially in the anterior commissure (-13.7%, p<0.001). Global brain atrophy seemed to be further aggravated at 30 weeks (-13.9%, p<0.05). At this time, atrophy was most severe in the parieto-temporal cortex (-20.8%, p<0.0001), frontal cortex (-19.8%, p<0.001), striatum (-19.8%, p<0.0001) and thalamus (-17.2%, p<0.0001), hippocampus (-15.1%, p<0.0001), motor cortex (-13.8%, p<0.0001). At 30 weeks, significant atrophy was also found in the midbrain (-18.8%, p<0.001) and brain stem (-16.9%, p<0.0001).

### Progressive metabolic impairment in the hippocampus of SCA7^140Q/5Q^ mice

**Fig 2:**
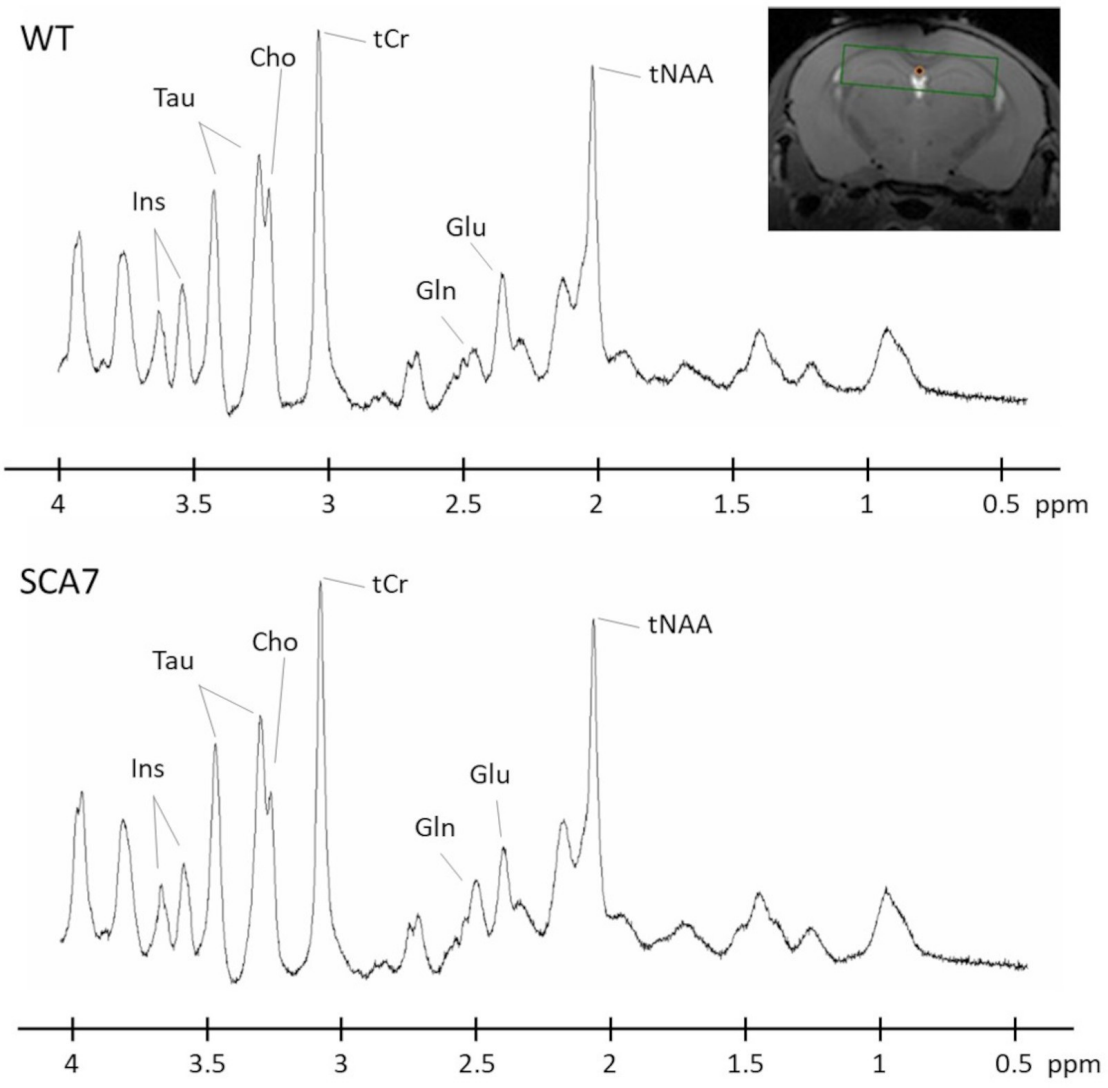
Representative spectra acquired in one mouse from WT (top) and SCA7 (bottom) groups. Spectra were acquired in a voxel located in the hippocampus (top-right corner). The following metabolites, total choline (tCho), total creatine (tCr), glutamate (Glu), glutamine (Gln), myo-inositol (Ins), total N-acetyl-aspartate + N-acetyl-aspartyl-glutamate (tNAA) taurine (Tau), and gamma-aminobutyric acid (GABA) were reliably quantified (CRLB < 5%).

The metabolic profile of both SCA7^140Q/5Q^ and WT mice was obtained by ^1^H-MRS on a voxel centered on the hippocampus, a structure that has been shown to be involved in the SCA7 pathophysiology [21]. Fig 2 shows representative spectra acquired at 16 weeks from the WT and SCA7 groups.

**Fig 3:**
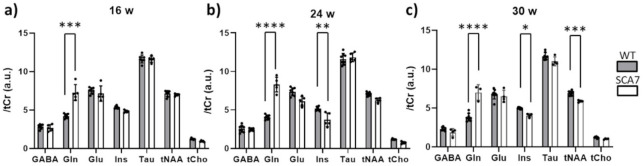
Evolution of metabolic profiles measured by ^1^H-MRS in the hippocampus of SCA7^140Q/5Q^ and WT mice. Quantification of metabolic profiles normalized to tCr level in WT (gray) and SCA7^140Q/5Q^ (white) mice measured at 16 (**a**), 24 (**b**) and 30 (**c**) weeks of age respectively. GABA = gamma-aminobutyric acid, Gln = glutamine, Glu = glutamate, Ins = myo-inositol, Tau = Taurine, tNAA = total N-acetyl-aspartate, tCho = total choline. Mean ± SD, n = 8 WT, n = 5 SCA7. *p<0.05, **p<0.01, ***p<0.001, ****p<0.0001 (Mixed model + Sidak’s multiple comparisons test).

Since the hippocampus showed atrophy at 24 weeks but not at 16 weeks, longitudinal analysis must determine whether metabolic alteration precedes or concurs structural alteration. The quantification of metabolic profiles acquired at different timepoints are shown in Fig 3. After normalization by tCr, metabolite quantification revealed a significant increase of Gln (+44%, p<0.001) in the hippocampus of SCA7^140Q/5Q^ mice at 16 weeks, while other metabolites showed similar concentrations in both groups. A progressive and significant increase of Gln was also measured in SCA7 mice compared to WT at 24 weeks (+76%, p<0.001) and 30 weeks of age (+83%, p<0.001). In addition, at these timepoints, Ins was significantly reduced in SCA7^140Q/5Q^ mice (24 weeks: -25%, p<0.05; 30 weeks: -16.4%, p<0.05). Finally, at 30 weeks of age, the tNAA level was also reduced (-16%, p<0.001).

## Discussion

By accurately translating the genetic and biochemical environment of human disease, transgenic and knock-in models can reproduce several phenotypes observed in polyQ diseases [8,22,23]. For instance, transgenic and knock-in mouse models of HD have shown excellent correlation with metabolic, structural, and microstructural impairments found in patients [15,24,25] and have been used to develop relevant non-invasive biomarkers [26,27]. Among animal models of polyQ diseases, heterozygous knock-in models expressing full-length mutant genes are highly valuable to mimic the presymptomatic stage of the disease. In this study, the longitudinal characterization of the SCA7^140Q/5Q^ mouse model provides for the first time significant insights into structural and metabolic brain alterations.

### Structural alterations suggest motor network impairments in the SCA7^140Q/5Q^ model

The longitudinal volumetric analysis based on anatomical imaging has revealed the progressive atrophy of multiple structures in the brain of SCA7^140Q/5Q^ mice. Atrophy worsens with time in two different ways. First, the volume of brain structures showing atrophy at early symptomatic stage (16 weeks) further decreases over time. Second, regions primarily intact show significant atrophy at later stages (24 and 30 weeks) and volume reduction extends to most brain structures by 30 weeks.

The automated atlas-based segmentation was used to extract the volume of brain regions without prior bias or operator interpretation. Interestingly, cortical regions, striatum and thalamus are among the earliest and most severely affected structures, losing nearly 20% of volume compared to wild-type mice. Atrophy of hippocampus is observed from 24 weeks, while the volume of midbrain and brain stem decreases at 30 weeks. Loss of grey matter tissue has been previously described in various brain regions in clinical SCA7, including the putamen and thalamus [28] using voxel-wise analysis. Diffusion tensor imaging has also demonstrated microstructural deficiencies in these structures [28]. Further investigation is needed to conclude on the nature of these defects, which can be attributed to either demyelination, neuronal death, or gliosis, as shown recently in a mouse model of Alzheimer’s disease [29]. In addition, both the striatum and thalamus are part of the sensory-motor network, which was found to be altered in SCA7 patients [30,31], as well as in HD in correlation with the severity of motor symptoms [32]. In SCA7, correlations were found between ataxia severity score of patients and impairment of several white matter tracts, including the anterior thalamic radiation [33]. Thus, the vulnerability of thalamus and striatum may reflect ataxia severity in the SCA7^140Q/5Q^ model.

### Metabolic modifications show hippocampal alteration in the SCA7^140Q/5Q^ model

^1^H-MRS can provide biological information occurring *in vivo* at the cellular level and is a powerful tool to identify relevant biological markers of the disease. Here, we evidence that metabolic profiles measured in the hippocampus of SCA7^140Q/5Q^ mice and their WT littermates are significantly different as early as 16 weeks. Profiles acquired at 3 different time points show a progressive disorganization of the hippocampus metabolism of SCA7^140Q/5Q^ mice. We measured a significant increase in [Gln] that precedes hippocampal atrophy, and which persists over time. As Gln is synthesized from Glu in astrocytes, this alteration suggests an early modification of the glial metabolism that may underly a problem with Gln transport [34]. Surprisingly, a decrease of [Ins] was measured at 24 weeks in SCA7^140Q/5Q^ mice. The interpretation of this decrease is not straightforward. It may reflect an impairment of osmotic regulation from astrocytes as already reported [35]. Alternatively, reduced levels of Ins in our SCA7 mice is reminiscent of that reported by Costa et al. in SCA3 models [36]. As suggested by Costa et al., this might indicate disturbances in phospholipid membrane metabolism and demyelination.

Interestingly, a significant decrease of [tNAA] was measured at 30 weeks in SCA7^140Q/5Q^ mice and may reflect a neuronal alteration. Indeed, tNAA is mostly located in the neuronal compartment [12], and its decrease has already been used as a marker of neuronal dysfunction [37]. The decreased level of tNAA observed in 30 weeks-old SCA7^140Q/5Q^ mice is in agreement with ^1^H-MRS studies in SCA7 patients [38]. The finding of metabolic impairments in both hippocampal neurons and astrocytes from SCA7^140Q/5Q^ mice lays the groundwork for investigating the underlying molecular mechanisms at the level of cell-type specific resolution.

In the present study, absolute quantification of metabolites was not possible, so metabolite levels were normalized to tCr values as classically performed in several ^1^H-MRS studies. As we did not observe an overall decrease in all metabolite levels in the SCA7 group over time, we ruled out a potential bias due to tCr normalization. Consequently, the decreases in tNAA and Ins measured at 30 weeks appeared to be related to the pathology. In consistence with the decrease of NAA, Glu levels tend to be lowered in the hippocampus of SCA7^140Q/5Q^ mice. However, this effect was not significant, probably due to small sample size, and deserves further investigation. The difference in size effect between changes in Glu and Gln could reflect the brain’s homeostatic response to maintain Glu uptake in neurons, possibly through *de novo* synthesis [39].

Recent studies showed altered functional connectivity between the cerebellum and hippocampus or parahippocampal areas of patients, which may account for specific memory dysfunctions [4,30,33]. Interestingly, hippocampal neurons show accumulation of mATXN7 and alteration of short-term synaptic plasticity in SCA7^266Q/5Q^ mice [7].

### Study limitations

Based on ex vivo images obtained in a previous study, the hippocampus appears to be one of the most affected structures [8], providing rationale for acquiring ^1^H-MRS spectra in a voxel located in this particular structure. In this study, the longitudinal *in vivo* measurements of brain structures volume showed that the thalamus and striatum are more severely atrophied than the hippocampus. While discrepancy between structure alterations as measured by *ex vivo* and *in vivo* volumetry is not fully understood, it may arise from brain structure alteration during tissue fixation [8]. This clearly illustrates the interest of longitudinal follow-up performed in the current study and indicates that it would be of great interest to henceforth acquire ^1^H-MRS metabolic profiles in these two particular structures. Nonetheless, the results obtained in the hippocampus are highly significant as this structure has been shown to be involved in the SCA7 pathophysiology [21].

Our results show several significant differences between SCA7^140Q/5Q^ and WT mice that were already present at the first timepoint of 16 weeks, in association with the onset of motor defects. However, mutant ATXN7 abnormally accumulates in neuronal nuclei already as early as 12 weeks. In the future and with the perspective of characterizing the prodromal phase of the disease, it would be relevant to acquire anatomical images and ^1^H-MRS profiles at early timepoints and evaluate the time window of appearance of brain structure and metabolic modifications.

The cerebellar vermis of SCA7^140Q/5Q^ mice is reduced in length and width, as measured by histology [8]. PC vulnerability is evident, with electrophysiological defect, cell morphometry alteration and dysregulation of gene expression when compared to wild type. In this work, the limited FOV coverage of the brain by our cryoprobe led to poor signal-to-noise ratio of the cerebellum. Therefore, the cerebellum could not complement our study of non-cerebellar structures. Changes highlighted in this paper may be consequences of the severe dysfunction of cerebellar Purkinje cells in SCA7, or may occur independently in other structures. Further comparative studies focusing on the cerebellum would be of great interest to better characterize this model.

## Conclusion

The present study exposes new data about the mouse model SCA7^140Q/5Q^, with longitudinal MRI and MRS characterization. Our results highlight a vulnerability of the striatum and thalamus in heterozygous mice, which could reflect an alteration of the sensory-motor network, as observed in the clinical form of SCA7. We observed variations in hippocampal metabolic profiles, which are generally interpreted as impairments of neuronal and astrocytic compartments. However, histological validations would be required to associate these variations with potential alterations of such brain cells. Our results suggest that dysregulation of the brain metabolism occurs in other structures than the cerebellar area and may play a crucial role in the development of the pathology, especially at early phases of the disease.

Our data provide a deep characterization of this model with temporal progression of the volume loss in different regions of the mouse brain, as well as information on the metabolism deficiencies occurring during the lifetime of SCA7^140Q/5Q^ mice. The results confirm that the SCA7^140Q/5Q^ model can be used to better understand the pathophysiology of SCA7 and highlight the potential of anatomical MRI and metabolic profile as measured by ^1^H-MRS as reliable biomarkers to monitor disease progression and for further evaluation of therapeutic treatments.
